# Musculoskeletal healthcare: Have we over‐egged the pudding?

**DOI:** 10.1111/1756-185X.13710

**Published:** 2019-11-13

**Authors:** Christopher G. Maher, Mary O'Keeffe, Rachelle Buchbinder, I. A. Harris

**Affiliations:** ^1^ Institute for Musculoskeletal Health Sydney NSW Australia; ^2^ School of Public Health The University of Sydney Sydney NSW Australia; ^3^ Monash Department of Clinical Epidemiology Cabrini Institute Melbourne Vic. Australia; ^4^ Department of Epidemiology and Preventive Medicine School of Public Health & Preventive Medicine Monash University Melbourne Vic. Australia; ^5^ South Western Sydney Clinical School Ingham Institute for Applied Medical Research Sydney NSW Australia

The phrase “to over‐egg the pudding” is a terrific analogy for what is now increasingly common in health care: medical overuse. Cooks would know that if you skimp with the eggs a pudding won't hold together and if you use too many eggs the pudding will go rubbery. And in musculoskeletal health care we also need to get the balance right. People's health can suffer when they receive too little health care and also if they receive too much health care.

The problem of too little health care is well recognized and it is easy to understand that patients’ health can be put at risk by underuse of proven healthcare services. However, the opposite problem is also possible but is less well recognized.^1^ In this editorial we adopt some perspectives from the field of overdiagnosis to consider overuse in musculoskeletal health care.

Overdiagnosis^2^ is an unwarranted diagnosis that leads to unnecessary treatments that do not benefit patients and that wastes health resources that could be better used elsewhere. Overdiagnosis also may cause harms: direct effects, unintended/indirect consequences, psychological impact, costs and resource implications, opportunity cost. We focus on 4 aspects of overdiagnosis that can lead to medical overuse in musculoskeletal health care:

- Overtesting where patients receive unnecessary tests
- Overdetection where clinicians act upon clinically unimportant findings
- Overdefinition where the boundaries between disease and health are shifted to encourage more healthcare, and
- Overtreatment where culture, industry and health systems encourage treatment with no net benefit.

We provide some examples that illustrate the nature and size of the problem,^3^ and highlight potential drivers of overuse of musculoskelatal health services (Table 1).

**Table 1:** Potential drivers of overuse of musculoskeletal health services

**Driver**	**Examples**	**Impact**
**Overtesting** Ordering unnecessary tests	Acting upon a single red flag to trigger diagnostic work‐up and/or specialist referral Frequent vitamin D testing	Up to 80% of patients with low back pain have at least one positive red flag Medical Benefits Scheme costs for vitamin D testing rose from $109.0 million in the 2009‐2010 financial year to $151.1 million in 2012‐2013^5^
**Overdetection** Clinicians act upon clinically unimportant findings	Incidental findings on imaging trigger unnecessary treatment Judging minor postural variations as abnormal triggers interventions to correct the abnormalities	Arthroscopic procedures for degenerative knee disease cost more than $3 billion per year in the USA. Medicalizing infancy by diagnosing notional spinal lesions that require manipulative care^31^
**Overdefinition** Changed disease boundaries encourage more health care	Promoting Pain as the Fifth Vital Sign encouraged treatment of any level of pain Disease subcategories that are no more than nominal diagnoses (eg instability) encourage use of ineffective therapies Creating the label “neuropathic” low back pain encouraged the use of pregabalin for low back pain	Contributed to the opioid crisis that has reduced life expectancy in the USA Spinal fusion is the most expensive surgical procedure in the USA (US$12.8 billion annually) There has been a surge in the use of pregabalin for pain and in parallel an increase in pregabalin poisonings, abuse and deaths
**Overtreatment** Culture, industry and health systems encourage treatment that does not provide a net benefit	There is a predisposition with regard to health care to believe that more is better and that new is better Professional associations encourage care for musculoskeletal conditions with a good natural history Health systems reimburse and/or commission more complicated care than is necessary	Proliferation of stem cell clinics offering treatments for musculoskeletal conditions resulting in high costs, direct and indirect harms Increased treatment rates based on belief of trusted sources (professional societies and individual professionals) Higher rates of procedures performed in regions where reimbursement is higher resulting in unwarranted practice variation

## 1 OVERTESTING

A common starting point for overdiagnosis in musculoskeletal health care is overtesting; where patients receive unnecessary tests. A good example is the uncritical interpretation of red flags to screen for serious pathology. Some texts and guidelines inadvertently encourage medical overuse by offering a long list of red flags and encouraging diagnostic work‐up and/or specialist referral if even a single red flag is positive. One study in Australian general practice found that of 1172 consecutive patients with back pain, 80% recorded a +ve response to at least 1 of the 25 red flags that were considered by the study general practitionerss.^4^ The irony here is that even though the clinicians were acting in good faith and aiming to help their patients, they may have harmed them through overdiagnosis. Other examples in musculoskeletal health care include repeat vitamin D testing; in Australia Medical Benefits Scheme costs for vitamin D testing rose from $109.0 million in the 2009‐2010 financial year to $151.1 million in 2012‐2013.^5^ In the sports medicine field it is common to hear of professional athletes who have sustained an acute hamstring muscle strain injury undergoing magnetic resonance imaging to guide management and predict return to sport, but neither is supported by robust evidence.^6^ The concern here is the possibility this practice leak may leak out into the wider community.

## 2 OVERDETECTION

In the overdiagnosis literature overdetection refers to the identification of abnormalities that resolve spontaneously or would not progress sufficiently to cause symptoms or harm during a person's lifetime.^7^ In the musculoskeletal field most incidental findings are picked up by overtesting in people with symptoms; using tests that commonly yield positive test findings in asymptomatic people. The challenge is then determining if the finding is relevant or not. A good example of medical overuse driven by overdetection would be acting upon the incidental findings commonly found with musculoskeletal imaging (eg lumbar disc degeneration, rotator cuff tear, femoroacetabular impingement, heel spur) and initiating more intensive treatment for the patient (eg specialist referral, surgery). What compounds the problem is that many of the surgeries that are encouraged (eg knee arthroscopy,^8^ subacromial decompression^9^) are now known to be no more effective than placebo. In all these cases the medical overuse is triggered by an unwarranted diagnosis.

Overdetection is not confined to the tests that would typically be considered the domain of the medical profession. In physiotherapy, podiatry and chiropractic, the treatments that characterize these professions are primarily driven by assessment of factors such as posture, range of motion, alignment, weakness, balance and coordination. A problem can arise if a clinician mistakenly judges a minor variation in one of these factors as abnormal, and institutes interventions to correct the presumed abnormality (eg sacroiliac dysfunction, lumbar instability, poor hip control). A somewhat related issue is the use of these types of tests to predict risk of injury in sports people. A battery of such tests, the Functional Movement Screen, has been recently studied and shown to perform no better than chance in predicting which professional soccer players would sustain an injury.^10^

## 3 OVERDEFINITION

Overdefinition encourages medical overuse by changing disease boundaries. This can happen by lowering the threshold that defines a disease or by expanding disease definitions.^7^ It has been suggested that before changing disease definitions it is necessary to consider certain issues. How many people will be affected? Why is the change necessary? Can the new disease label be reliably used with patients? What is the balance of benefits and harms for patients and society with the change?^11^ In musculoskeletal health care there are many examples where disease boundaries have been changed and the challenge is distinguishing when this change has led to medical overuse, patient harm and waste; and where the change has provided benefits to patients and society.

The initiative “Pain as the Fifth Vital Sign” lowered the threshold for medical treatment of pain to any pain score >0. This change has been suggested to be one of the important drivers of the current opioid epidemic which has claimed hundreds of thousands of lives.^12^ However, it is important to recognize that the Pain as the Fifth Vital Sign initiative was motivated by compassion for patients and it was events happening in parallel where opioid medicines were misleadingly marketed to doctors, patients and patient advocacy groups that enabled overdefinition to cause so much harm.

Another way to change disease boundaries is to subdivide broad non‐specific disease categories into subcategories that are more targeted and precise. For example, some in the back pain field dismiss the label non‐specific low back pain and instead argue for labels targeting a specific structure (disc, facet joint) or mechanism (eg instability) and the use of similarly targeted personalized therapies. The problem is that the diagnoses offered are nominal diagnoses^13^ that drive more invasive, costly and ineffective therapies without providing benefit.^14^, ^15^ The resulting overuse is substantial: for example the most expensive surgical procedure (US$12.8 billion per annum) in the USA is spine fusion, a procedure that is most commonly performed for degenerative conditions for which there is good evidence of harm and poor evidence of benefit over cheaper and safer alternatives. Also in the USA, there are nearly 1 million facet joint injections and half a million neurotomies performed annually at great cost despite good evidence showing lack of effectiveness.^16^ The specific diagnoses also provide a target site for injections of stem cells, blood products and sclerosing agents which are unproven, expensive and have the potential for harm.^16^

“Central sensitization” and “nociplastic” pain are both nominal diagnoses which have arisen out of attempts to explain (and eventually treat) painful conditions but both have the potential to drive medical overuse because there is no evidence that the label leads to cost‐effective treatment that provides more benefit than harm. The labels sarcopenia and osteopenia are normal aspects of aging (especially when defined relative to young people) but they have now become diseases to be prevented and treated. These are good examples of changing disease boundaries as they blur the line between disease and aging. While the desire to reverse or prevent undesirable aspects of aging is understandable, these diagnoses have not been shown to provide a net benefit to “sufferers”.

An example of expanding disease definitions was borrowing the “neuropathic pain” label, typically used for less common conditions such as post‐herpetic neuralgia, and attaching it to a much more common condition: low back pain. The new diagnosis “neuropathic low back pain” and its potential treatment with gabapentionoids was promoted to clinicians and reinforced by a consumer‐facing disease awareness program. This led to a marked increase in the use of pregabalin in many countries.^17^ Subsequently we have learnt that pregabalin is ineffective for sciatica and low back pain,^18^ but is associated with serious harms including death and dependence.^19^

Broadening disease boundaries is not always harmful and in some situations, it is yet not clear. Non‐radiographic axial spondyloarthritis is an example of lowering the threshold that defines a disease, in this case by removing the requirement for imaging evidence of the condition. At present the balance of benefits and harms is unclear. However, this change has arguably benefited some patients because it opened access to cost‐effective therapies; but it may have caused societal harm through leakage of very expensive biological treatments to people with non‐specific low back pain who have been labeled as having non‐radiographic axial spondyloarthritis.

## 4 OVERTREATMENT

In an ideal healthcare system all patients would receive the right care, that is “care that is tailored for optimizing health and wellbeing by delivering what is needed, wanted, clinically effective, affordable, equitable, and responsible in its use of resources”.^1^ However, culture, industry and health systems can disrupt this approach and encourage overtreatment where patients are provided with treatments that do not provide a net benefit.

Musculoskeletal health care has a rich history of innovative tests and treatments escaping into routine care before we completely understand the balance of benefits and harms. A recent example is autologous stem cell interventions^20^ which have grown in popularity in many countries and tend to focus on treatment of musculoskeletal conditions. For example a 2018 study of 432 US businesses engaged in direct‐to‐consumer marketing of stem cell interventions found that 387 businesses offered services for musculoskeletal conditions (orthopedic conditions, pain relief, sports injuries).^21^ These high‐cost interventions have not been shown to be effective but have been associated with significant harms.

The premature uptake of unproven therapies can in part be explained by prevailing beliefs. Clinicians and patients both tend to overestimate benefits and underestimate harms of tests and treatments.^22^, ^23^ This may reflect the cognitive biases that we are all susceptible to^24^ but is also likely to be influenced by inaccurate health information that clinicians and patients are exposed to. News stories related to health interventions can be inaccurate,^25^ with a key problem being exaggerated health news,^26^ and interestingly this seems to be at least partly driven by exaggeration in the academic press releases that initially prompted the news story.^27^, ^28^ Health information developed for consumers can also be lacking. A study of Australian consumer information on knee arthroscopy for symptomatic osteoarthritis found that only 6 of 93 documents cited research and only 8 of 93 advised against the procedure.^29^ A study of information on low back pain on 79 “trustworthy” (eg government, university, hospital) websites from six countries found that less than the half the treatment recommendations were accurate.^30^ There is also predisposition with regard to health care to believe that more is better and that new is better.^3^

Many professional associations have marketing and advocacy programs directed to the general public; but if these encourage care for conditions with a good natural history, for example non‐specific low back pain and ankle sprains, there is a substantial risk of overtreatment. In the USA initiatives such as GetPT1st, ChoosePT and PTNow market the concept that early physical therapy is vital for recovery from musculoskeletal conditions and there is little or no mention of the option of self‐management and staged or stratified care.

Health systems can drive overtreatment through the approaches taken to regulation, reimbursement and commissioning of health services. The end result can be that patients may receive more complicated care than is necessary (eg image‐guided steroid injections at sites where blinded injections are just as effective, inpatient rehabilitation vs home rehabilitation following knee replacement which is just as effective, robotic surgery) or care that is ineffective (eg arthroscopy for knee osteoarthritis, spinal fusion surgery) or of unknown value (eg stem cell injections). In Australia, while no longer the case for new items, the Pharmaceutical Benefits Scheme and Medical Benefits Scheme reimburse or subsidize ineffective treatments for chronic musculoskeletal pain (eg spinal surgery, pregabalin, opioids) but not treatments that are endorsed in guidelines (eg psychological pain management).

## 5 CONCLUDING REMARKS

In musculoskeletal health care overdiagnosis is widespread and leads to unnecessary tests and treatments that do not benefit patients, and may cause harms, and waste health resources that could be better used elsewhere. While the media often portrays medical overuse as being deliberate, driven by greed and dishonesty, often medical overuse is well intentioned. Overuse has many potential drivers, including educational deficiencies, commercial incentives and reluctance to challenge the status quo.

We see there are two important steps that need to take place to address the issue. The first is that there is a clear need to inform consumers, clinicians, decision makers and the public about the extent of, and consequences of, overdiagnosis‐driven medical overuse in musculoskeletal health care. In parallel we need a research program to characterize the problem, identify causes and develop responses to address it.^2^
